# Successful Outcome in an Operated Case of Small Bowel Obstruction: Unmasking the Culprit

**DOI:** 10.7759/cureus.46507

**Published:** 2023-10-05

**Authors:** Mahak Choudhary, Kamlesh Chaudhari, Sanket S Bakshi

**Affiliations:** 1 Department of Obstetrics and Gynecology, Jawaharlal Nehru Medical College, Datta Meghe Institute of Higher Education and Research, Wardha, IND; 2 Department of Medicine, Jawaharlal Nehru Medical College, Datta Meghe Institute of Higher Education and Research, Wardha, IND

**Keywords:** resection, mitotic index, anastomoses, bowel obstruction, leiomyoma

## Abstract

Abdominal distension, constipation, and vomiting are just a few of the symptoms of small bowel obstruction (SBO), a disorder with several well-known frequent causes. Patients may now be more carefully chosen for surgical intervention and frequent causes of SBO can be quickly detected thanks to recent advancements in both imaging modalities and minimally invasive procedures. Despite these developments, it must be emphasized that diagnosing unusual causes of SBO remains challenging. This study describes a 38-year-old female patient who was diagnosed with a capsulated submucosal leiomyoma and later treated surgically.

## Introduction

The benign smooth muscle tumors known as leiomyomas (fibroids) develop in the uterine myometrium. They can result in considerable morbidity, such as excessive menstrual bleeding and pelvic pressure, and are symptomatic in 25% of individuals, whereas the prevalence of bowel obstruction in the adult population ranges around 5% [1,2]. We report a patient with a small bowel obstruction (SBO) brought on by a big uterine fibroid in this case study. Fibroid complications are uncommon. For symptomatic individuals, surgery continues to be the basis of therapy. With a protracted small bowel obstruction, the gut continues to dilate, raising the luminal pressure and increasing the risk of intestinal ischemia, perforation, peritonitis, and sepsis [3,4]. As this pressure keeps increasing, it surpasses the capacity of the local venous drainage system, which causes intestinal edema and hyperemia [5]. As the pressure continues to rise, the vascular supply to the colon is compromised, which leads to ischemia and ultimately to perforation [6].

## Case presentation

After initially stabilizing, the patient was referred to the gynecology department, where a repeat general examination revealed a high body mass index (BMI) of 29.3 and that she was afebrile. The 38-year-old female having parity of two and no further fertility wishes, had complained of diffuse lower abdominal pain for two days that had worsened and been accompanied by several episodes of vomiting; her last bowel movement had been 72 hours prior. A palpable, painful abdominal mass was seen in the lower abdomen during an abdominal examination. Due to the patient's obesity, a vaginal examination was not possible, but a digital rectal examination revealed firm stools with no blood. As shown in Table 1 arterial blood gas analysis revealed that serum plasma values were deranged.

**Table 1:** Deranged plasma values.

Serum parameter	Actual value	Reference range
White blood cell count	12.4 x 10^5^/mL	4.0-11.0 x 10^5^/mL
Hemoglobin	10.0 g/dL	12.0-17.0 g/dL
C-reactive protein (CRP)	15 mg/dL	0.3-1.0 mg/dL
Na^+^	122 mEq/L	135-145 mEq/L
K^+^	4.9 mmol/L	3.5-5-5 mmol/L
Urea	11.7 mmol/L	2.1-8.5 mmol/L
Creatinine	125 µmol/L	50-98 µmol/L
Platelets	1.6 x 10^5^/µL	1.5-3.0 x 10^5^/µL

**Figure 1:**
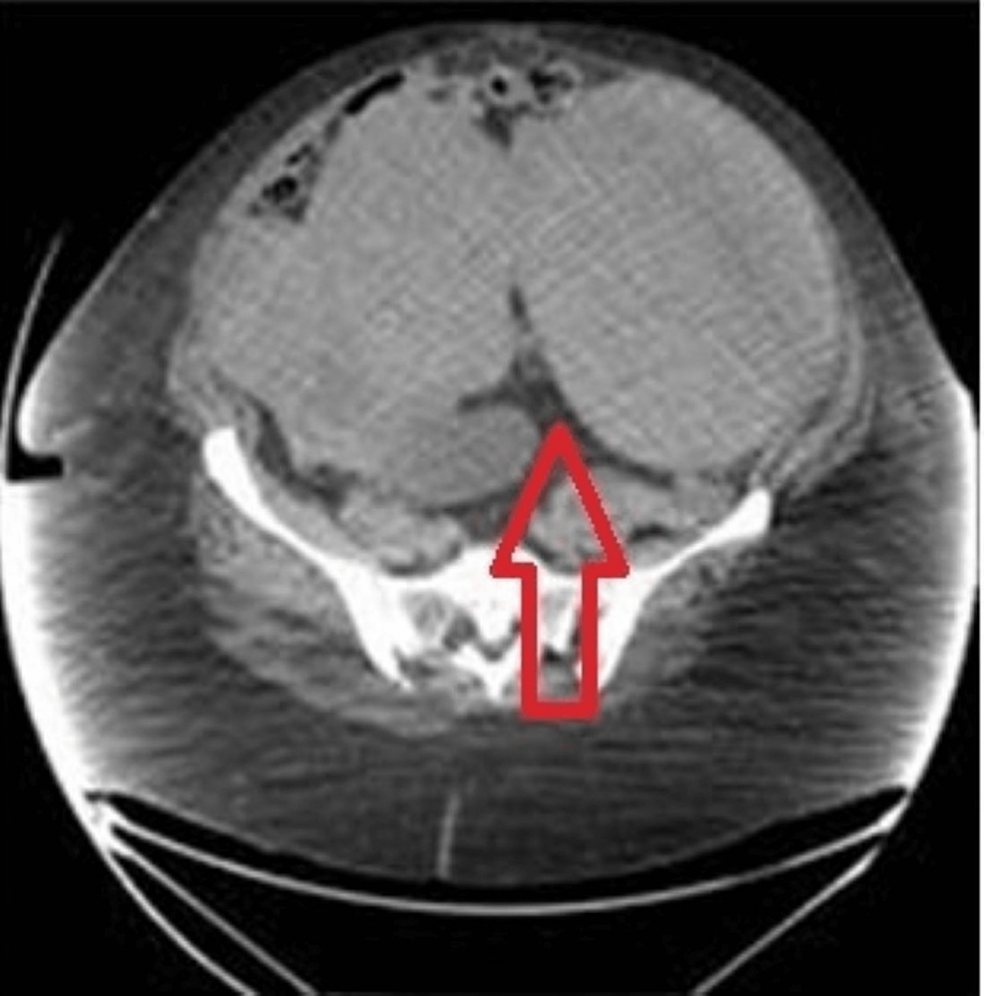
Computed scan showing a lobulated abdominal mass.

An abdominal computed tomography scan revealed a big, lobulated mass that may have been a fibroid that measured 19 × 15 cm and was made up of heterogeneous endometrial tissue with a focal region of calcification as shown in Figure 1. The small bowel loops were dilated, which is a sign of a small intestine obstruction. Intravenous antibiotics and saline resuscitation were administered first. For bowel relief, we placed a nasogastric tube, as it helps in bowel decompression.

**Figure 2:**
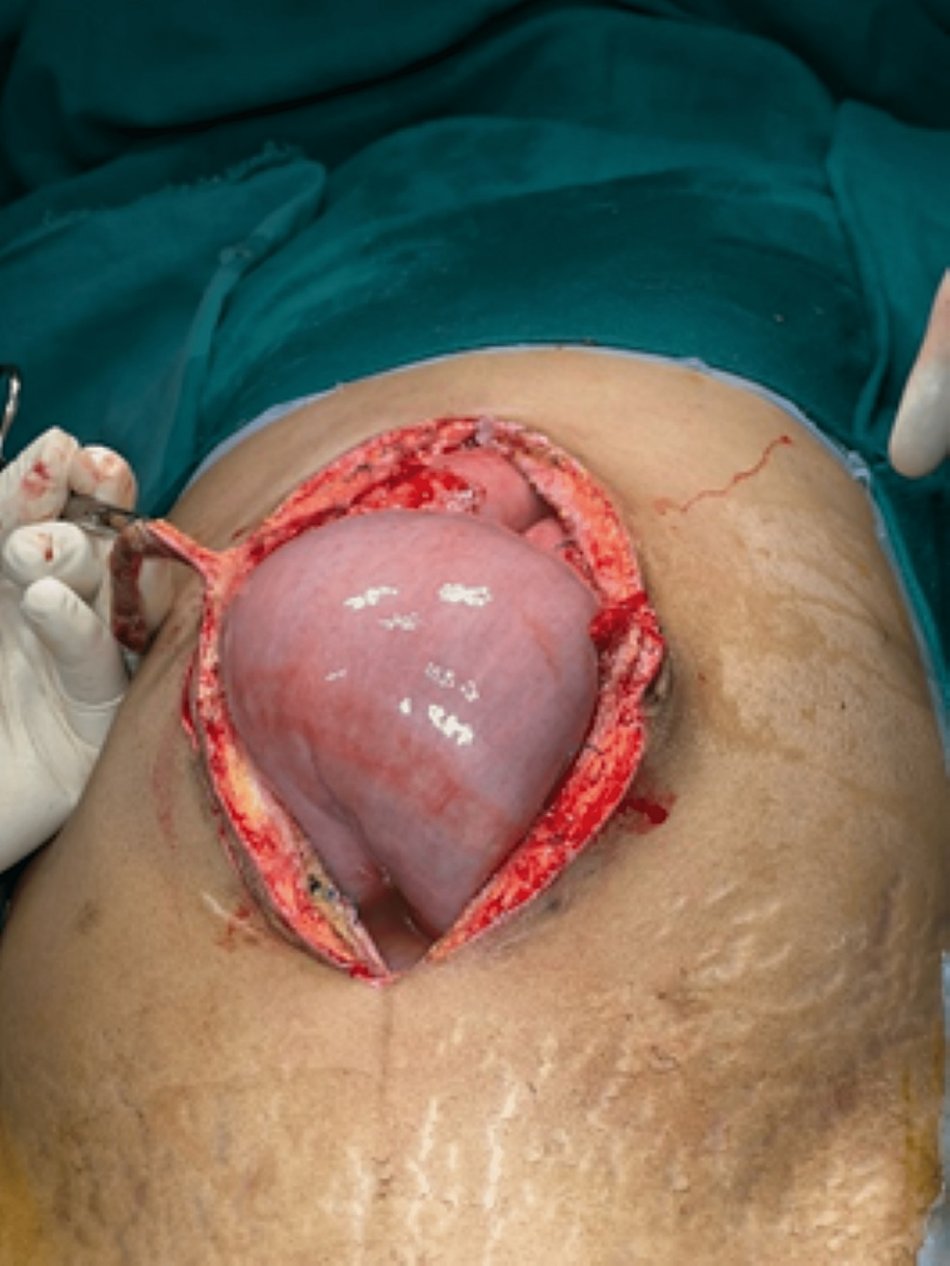
Dilated loop noticed on laparotomy.

**Figure 3:**
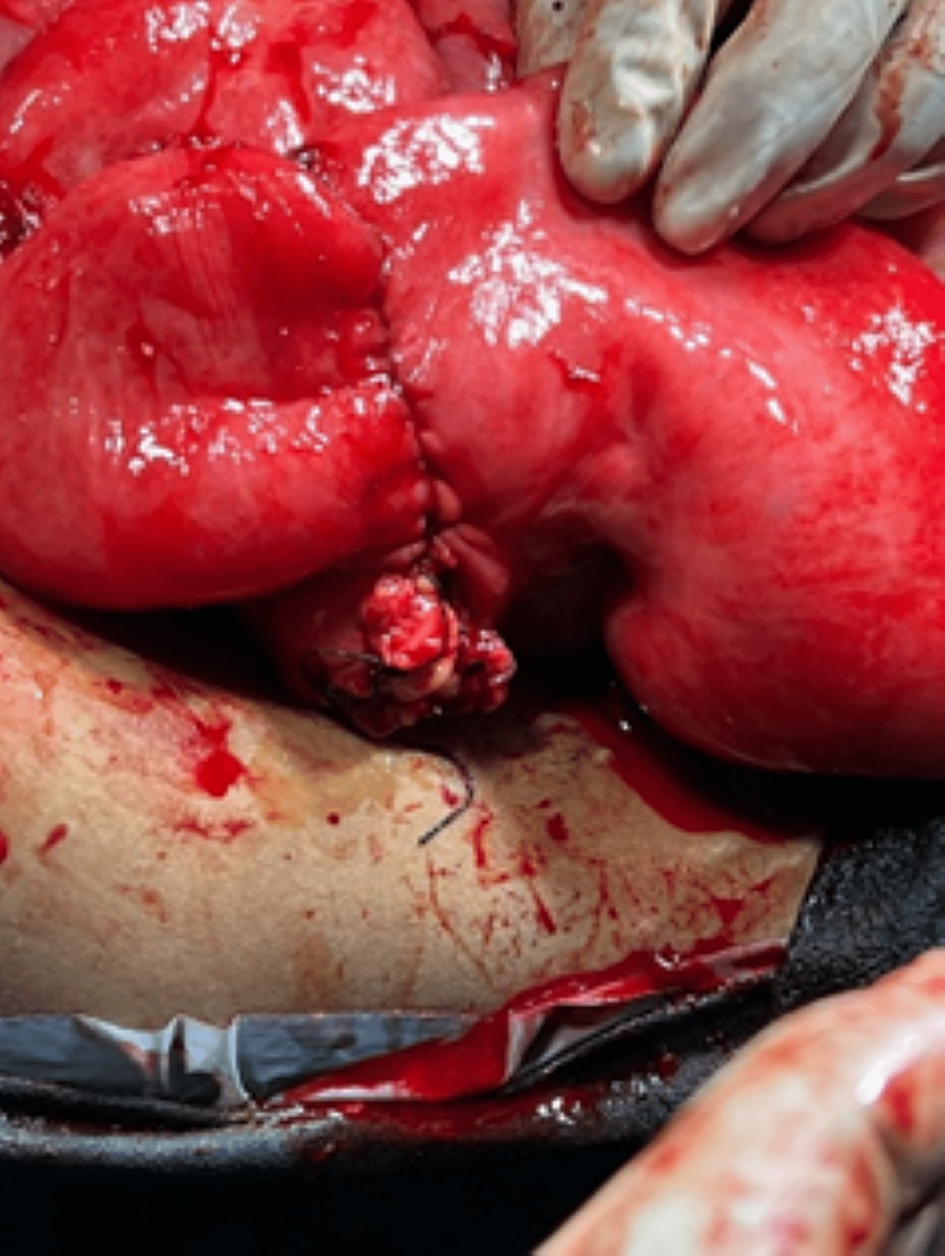
Anastomoses were carried out and the incision was closed with 2-0 nylon suture.

**Figure 4:**
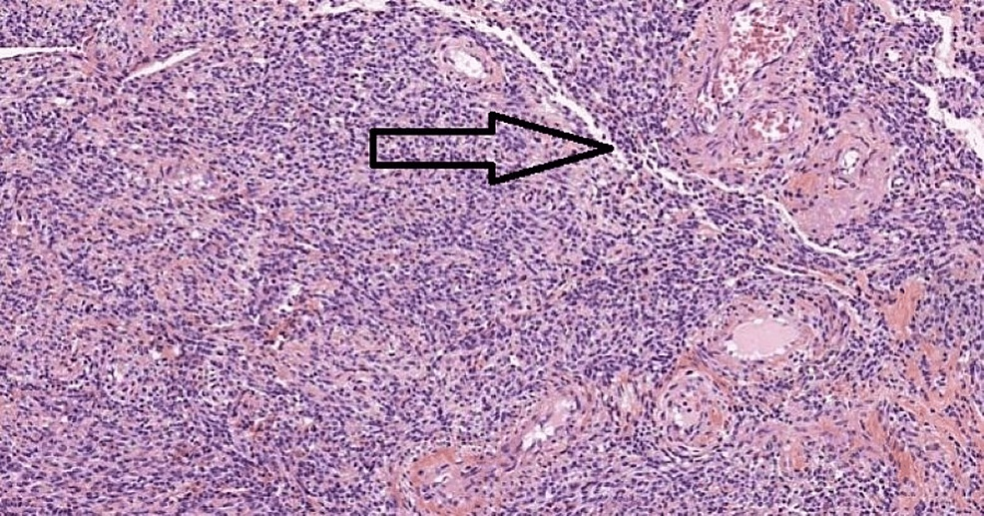
Histopathology section showing increased mitotic index, cellular atypia, and capsulated mass.

Due to the escalating stomach discomfort the day after admission, we chose to do an abdominal hysterectomy. The surgical management options were discussed with the patient beforehand, taking into account that the patient did not wish to conceive in the future. The patient was placed in a supine position and was administered with general anesthesia. A midline skin incision was made on the abdominal wall. We discovered a significantly dilated loop with a palpable mass following the dilatation 25 cm proximal to the mid-section of the jejunum and with limited intraperitoneal serous fluid as depicted in Figure 2. This portion, which included the lump, was removed and sent for pathological examination. Further after developing a clear visual field, the round ligament was ligated and cut. The fallopian tubes along with the ovarian ligament were ligated and cut. After mobilization of the bladder and successful amputation, the anastomosis was completed with staples with 2-0 basic nylon sutures, the wound was stitched up as shown in Figure 3. The submucosal leiomyoma measured 19 cm in size and the histopathological findings have been presented below in Figure 4. Postoperative period was rather uneventful. On the sixth day following surgery, we released her without any issues. She arrived at the outpatient clinic for follow-up 15 days after being discharged with no symptoms, the ability to consume more food, and regular bowel movements.

## Discussion

Small bowel obstruction (SBO), a notorious clinical complication, can have a variety of causes depending on the patient's age, with benign illness typically being the culprit in children and adolescents whereas malignant or adhesive disease is much more prevalent in older individuals [7,8]. In fact, it has been demonstrated that over 50% of instances of intestinal blockage are caused by adhesions [5]. First-line imaging for suspected bowel obstruction is advised to include an abdominal radiograph to determine the anatomical level of the blockage and an erect chest radiograph to rule out perforation [3,9]. To determine the location and nature of the blockage and to make plans for subsequent care, computed tomography (CT) scanning is advised [10]. The most sensitive method for assessing the presence of peritoneal free air and the precise site of a particular hole is computed tomography imaging. However, CT is less sensitive for identifying the presence of ischemia. Therefore, CT imaging is helpful in the diagnosis and treatment of blockage on a variety of levels.

Bowel blockage is known to be caused by gynecological disorders and their treatments, but there is very little information on a particular instance, like the one described here. Although very infrequently, there is a connection between leiomyomas and intestinal blockage [7]. The problems of leiomyomas were previously described in a case report involving a big uterine leiomyoma and intestinal obstruction brought on by a cecal volvulus. Also possible is the formation of adhesions by leiomyomas, which might result in little or severe intestinal blockage [11]. Given their large size or as a result of the difficulties they might cause, uterine leiomyomas obviously have the potential to compress the intestine, causing blockage, as demonstrated by our case and another case report that was only recently published [12]. It is critical to examine leiomyomas as the cause of intestinal blockage in female patients, whether they have previously received a diagnosis or not.

The location, nature, and size of the mass are typically connected to the clinical presentation. A substantial number of asymptomatic instances have led to transvaginal ultrasound imaging being regarded as the first and most sensitive diagnostic method for this illness, even if the history and physical examination provide excellent guidance for the diagnosis. Regarding intestinal obstruction, CT scanning is preferable due to its benefit of pointing to the blockage's root cause, in this example, fibroids. The management protocol for such instances where incidental leiomyoma leads to bowel obstruction includes protocols such as nasogastric decompression. Additionally, analgesic medications are administered as part of the treatment [2,11]. The surgical measures to relieve the complications include procedures of adhesiolysis followed by myomectomy, in particular cases. The prognosis of this clinical pathology may take a worsening turn, if not given timely treatment.

## Conclusions

Huge uterine fibroid should be listed among the reasons for mechanical small intestine obstruction because it is an uncommon cause of intestinal obstruction. Radio imaging techniques, mainly computed tomography in such cases, have a vital role in diagnosing and deciding the further management plans. By preventing needless surgical intervention and lowering morbidity and death rates, accurate monitoring and imaging can improve patient outcomes. The treatment regimens include medical as well as surgical methods. The efficacy of both of the therapeutic regimens has been proven to be good, in which surgical procedures are the last go-to option if the mere medical treatment options do not work out. The accuracy of these surgical methods has been proven to be highly efficient and rather revolutionary prognostic benefits have been observed.
